# A Rapidly Growing Desmoid Tumor of the Anterior Chest Wall in a Young Woman: A Case Report

**DOI:** 10.7759/cureus.98170

**Published:** 2025-11-30

**Authors:** Juan J Villamarín, Ana M Rendón-Garavito, Andres Francisco Vasquez Perdomo, Angela Moreno

**Affiliations:** 1 Department of Radiology, Universidad de La Sabana, Chía, COL; 2 Department of Radiology, Fundación Santa Fe de Bogotá, Bogotá, COL

**Keywords:** breast, breast implants, clinical case report, desmoid tumors, rib resection, surgical case reports

## Abstract

Desmoid tumors (DTs), or aggressive fibromatosis, are rare fibroblastic neoplasms of connective tissue origin. They are locally invasive with a high rate of recurrence, although metastasis is rare. Their clinical behavior varies widely, as some tumors exhibit progressive growth, while others remain stable or may spontaneously regress. Symptoms depend largely on tumor size and location.

We present the case of a 25-year-old woman who developed a painful, enlarging mass in the right breast region over two months. Imaging revealed a 100 × 60 mm lesion involving intrathoracic structures and musculature, located posterior to a breast implant. Core needle biopsy confirmed an extra-abdominal DT of the anterior chest wall. Taking into account the symptom severity and rapid tumor progression, the patient underwent surgical resection with chest wall reconstruction using polypropylene mesh and titanium rib prostheses. Postoperative imaging showed complete resection, and the patient remained free of recurrence at two, four, and five years of follow-up. While conservative management with surveillance is increasingly recommended for stable DTs, surgery remains the primary treatment for symptomatic or fast-growing tumors, although new non-invasive methods are on the rise. Accurate diagnosis requires a combination of imaging and histopathological confirmation, and long-term follow-up is essential because of the risk of recurrence. This case highlights that early and complete surgical management, combined with appropriate reconstruction, can lead to favorable long-term outcomes and improved patient quality of life.

## Introduction

Desmoid tumors (DTs), also known as aggressive fibromatosis, are rare fibroblastic neoplasms that originate from connective tissue. While they exhibit locally invasive growth and tend to recur, they do not metastasize [1-3]. 

The incidence of DTs is low, with an estimated two to four cases per million person-years, making disease awareness and diagnosis particularly challenging [2,4]. DTs can arise in association with familial adenomatous polyposis (FAP) or on a sporadic basis. While FAP-associated cases result from germline mutations in the adenomatous polyposis coli (APC) gene, sporadic cases usually follow from somatic mutations in the CTNNB1 gene, leading to aberrant β-catenin accumulation [1,3,5]. DTs can develop in various anatomical regions, including the abdominal wall, intra-abdominal sites, and extra-abdominal locations such as the limbs and thoracic wall, as demonstrated in our case [3,5,6]. In particular, DTs are present in the chest wall in 8%-10% of all cases [7]. 

DT symptoms depend on their location and size. Patients may have pain, swelling, or functional impairment; extra-abdominal tumors can limit joint mobility, while intra-abdominal tumors might cause bowel obstruction. The course of the disease is erratic; some tumors show increasing growth, while others remain stable or even regress on their own [1,6]. 

This report describes the case of a young woman who developed a rapidly expanding mass in her right pectoral region, for which imaging studies were conducted and a biopsy was performed, resulting in the diagnosis of an anterior thoracic wall DT.

She was evaluated by the thoracic surgery team, who considered surgical management the best therapeutic option due to her marked symptoms. The operation was carried out without complications, and follow-up imaging showed no tumor recurrence. Thus, the tumor resection was deemed successful.

## Case presentation

**Figure 1:**
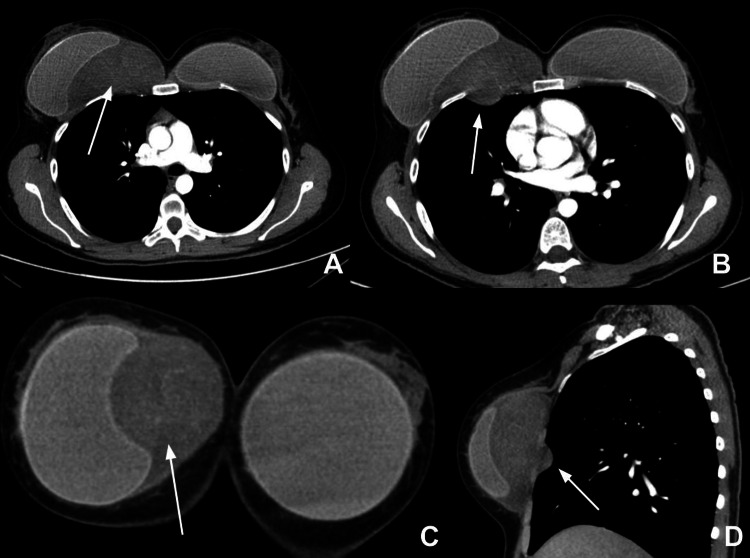
Chest computed tomography with contrast: Axial sections (A and B) show a round hypodense lesion with linear contrast enhancement with mild invagination towards the ipsilateral pulmonary parenchyma (arrows). Coronal reconstruction (C) shows displacement and deformity of the right breast prosthesis without infiltration (arrow). Sagittal reconstruction (D) confirms the posterior location of the lesion in relation to the implant and pulmonary invagination without capsular or adjacent tissue compromise (arrow).

A 25-year-old woman with a relevant medical history of appendectomy, cesarean section, and breast augmentation presented with a two-month history of a progressively enlarging and painful mass in the right pectoral area. Given these symptoms, a computed tomography (CT) scan of the chest, abdomen, and pelvis was requested as a diagnostic study, which revealed a solid lesion with lobulated margins, located posterior to the breast implant, containing vascular structures, measuring 100 × 60 mm in diameter, with intrathoracic and muscular involvement (Figure 1). 

**Figure 2:**
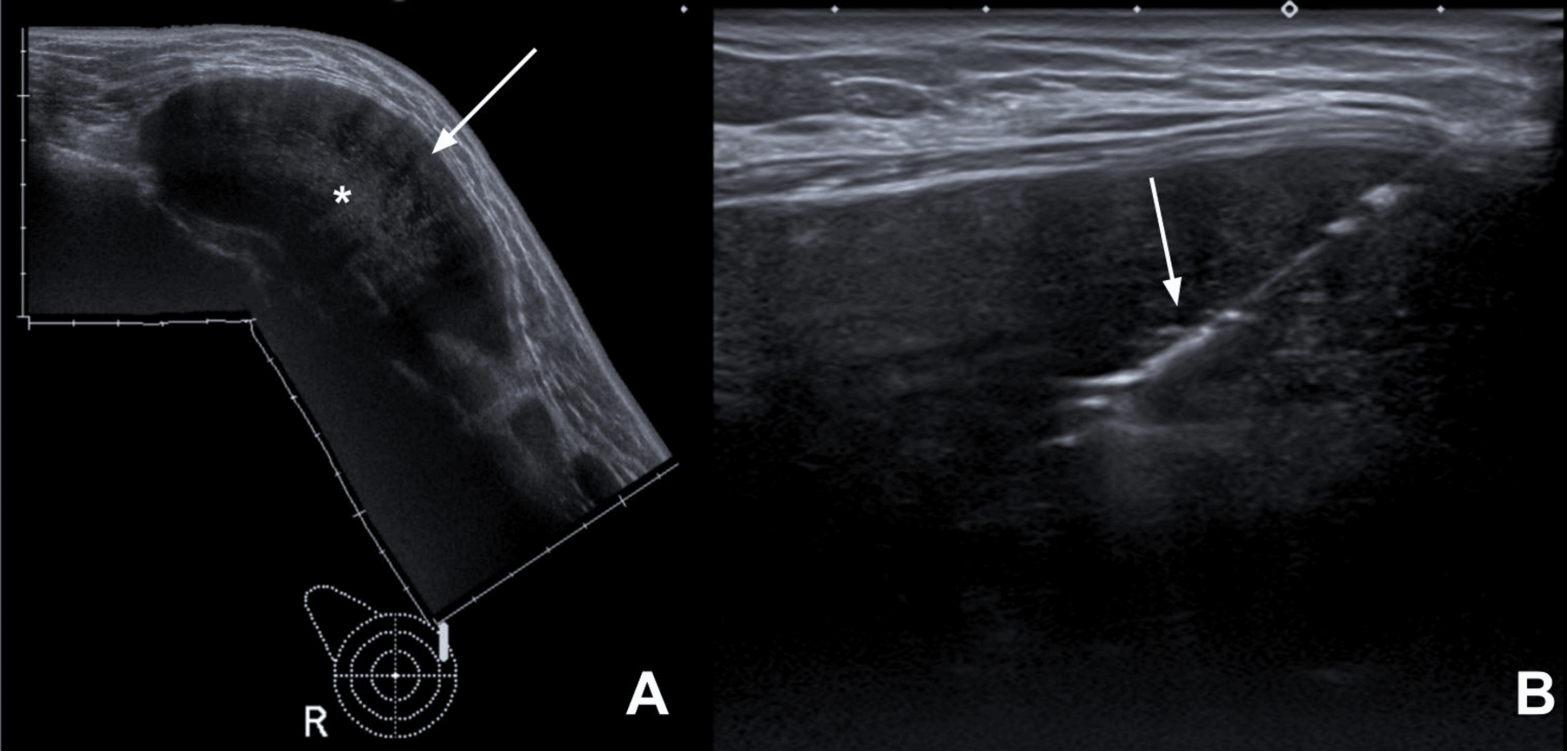
(A) Panoramic ultrasound image demonstrates a heterogeneous lesion located in the posterior region, medial to the breast prosthesis in the right rib cage. The lesion is predominantly hypoechoic (arrow) with a centrally located echogenic component (*). (B) A guided biopsy of the lesion was performed (trucut needle shown with the arrow).

A core needle biopsy confirmed the lesion to be an extra-abdominal DT situated in the anterior right thoracic wall (Figure 2). These results led to the patient's referral to the thoracic surgery department, where it was concluded that surgery was necessary to treat the tumor.

On preoperative physical examination, a deformity in the anterior right thoracic wall was noted due to a large, rigid, and painful mass. After preoperative evaluation and review of diagnostic imaging, the patient underwent surgery. A wide resection of the tumor from the anterior thoracic wall was followed by reconstruction using a polypropylene mesh and a titanium prosthesis for the third and fourth ribs to prevent pulmonary herniation. Postoperative chest X-ray, following surgery, revealed that the prosthesis was well placed and that the lesion had been resected entirely.

**Figure 3:**
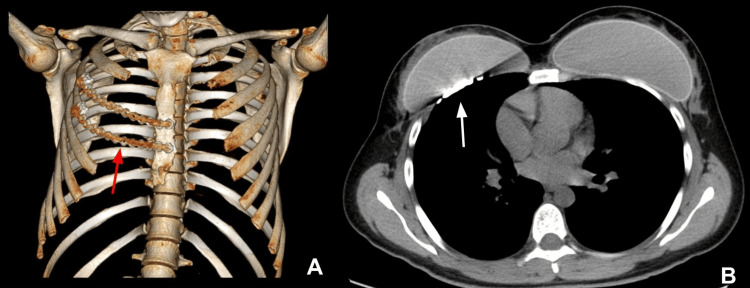
(A) Three-dimensional reconstruction of the rib cage demonstrates a rib prosthesis (red arrow) in the right hemithorax. (B) Postoperative control computed tomography following resection of the mass in the right rib cage shows complete removal of the previously described lesion with no evidence of suspicious residual or recurrent lesions at the surgical site. Osteosynthesis material is visualized within the rib cage (white arrow).

The patient remained hospitalized for five days for postoperative monitoring, then was discharged for outpatient follow-up. Seven months after surgery, a control chest CT revealed normal postquirurgical changes with total recovery and without the need for a second intervention (Figure 3). Two years later, and also in the fourth and fifth postoperative years, annual control CT scans were performed; no abnormality was found.

## Discussion

DTs are uncommon fibroblastic soft-tissue tumors characterized by locally aggressive growth and infiltrative behavior. These tumors can cause significant morbidity due to surrounding tissue compromise. In the general population, DTs affect two to four million individuals annually. However, in patients with FAP, in whom the APC gene is mutated, the risk of DTs has been estimated to be approximately 1000 times greater [2,4].

Diagnosis of DTs is a multiple-step process that includes history taking, physical examination, imaging studies, and microscopy of a biopsy specimen. The definitive diagnosis is made by histopathological assessment of the tumor tissue. Immunohistochemical analysis can help by demonstrating nuclear overexpression of β-catenin in the tumor cells; however, this marker is not specific for DTs and may not be present in every case. Genetic analysis can be more accurate, especially if it reveals mutations in the CTNNB1 or APC genes, which are more often linked to DTs. Imaging techniques can vary depending on the tumor’s location. For tumors in the abdomen, CT is usually preferred, while magnetic resonance imaging (MRI) is usually applied for tumors located outside the abdomen. Ultrasound is generally the method of choice in pregnant women, when the tumor is located in the abdominal wall or within the limbs [2].

The main differential diagnoses of DTs include other myofibroblastic diseases, such as sarcoma, gastrointestinal stromal tumor, and leiomyoma, with the most challenging differentials being nodular fasciitis and low-grade fibromyxoid sarcoma. It is noticeable that between 30% and 40% of DT cases are incorrectly diagnosed after histological examination [2]. MRI is the preferred radiologic study for follow-up. It is usually requested first in short intervals of one to two months, followed by intervals of three to six months. During follow-up, it is estimated that 50% to 60% of DTs remain stable after diagnosis, while 20% to 30% may regress, with some even completely resolving following an initial phase of growth [8].

Surgery used to be the treatment of choice, particularly in symptomatic cases or cases of rapid tumor growth, such as the tumor in our case [9-11]. However, as the recurrence of the lesion is high and surgical morbidity is a risk, conservative therapy is often employed. In many cases, active surveillance, accompanied by a proper radiological assessment, is recommended, especially for asymptomatic or stable tumors [1,4,8]. For tumors that are symptomatic or progressing, systemic therapies such as tyrosine kinase inhibitors or cytotoxic chemotherapy may be considered. There are new treatments, like γ-secretase inhibitors, which have potential but need further clinical trials to prove efficacy [1,2,5].

Prognosis is extremely variable, depending on the location of the tumor, the size of the tumor, and the response to treatment. Though DTs are not characteristically fatal, they may have a significant impact on quality of life due to pain and functional impairment [2,12]. Hence, multidisciplinary management and individualized treatment approaches are crucial for maximal outcomes in patients [1,5]. 

## Conclusions

DTs are uncommon, locally aggressive neoplasms that present significant diagnostic and therapeutic challenges due to their unpredictable clinical course. This case highlights the importance of a complete and comprehensive diagnostic approach, combining imaging, histopathology, and clinical evaluation, paired with surgical management. Successful outcomes can be achieved through a multidisciplinary approach and long-term follow-up, as the risk of recurrence still exists. Future research into targeted therapies may provide more individualized, less invasive treatment options, improving both disease control and quality of life.
